# Nasal *Staphylococcus aureus* in COVID-19 Patients Shows No Enrichment of High-Risk Clones

**DOI:** 10.3390/ijms27031250

**Published:** 2026-01-27

**Authors:** Lidia Piechowicz, Agnieszka Necel, Katarzyna Kosznik-Kwaśnicka, Magdalena Pałys, Anna Pałubicka, Jacek Międzobrodzki, Maja Kosecka-Strojek

**Affiliations:** 1 Department of Medical Microbiology, Faculty of Medicine, Medical University of Gdansk, Debowa 25, 80-204 Gdansk, Poland; agnieszka.necel@gumed.edu.pl (A.N.); katarzyna.kwasnicka@gumed.edu.pl (K.K.-K.); 2 Department of Laboratory and Microbiological Diagnostics, Specialist Hospital in Koscierzyna Sp. z o.o., Koscierzyna, Alojzego Piechowskiego 36, 83-400 Koscierzyna, Poland; magdalena.palys1@gmail.com (M.P.); apalubicka@op.pl (A.P.); 3 Department of Microbiology, Faculty of Biochemistry, Biophysics and Biotechnology, Jagiellonian University in Krakow, Gronostajowa 7, 30-387 Krakow, Poland; jacek.miedzobrodzki@uj.edu.pl (J.M.); maja.kosecka-strojek@uj.edu.pl (M.K.-S.)

**Keywords:** *Staphylococcus aureus*, virulence genes, spa-typing, COVID-19

## Abstract

*Staphylococcus aureus* nasal carriage is a potential source of secondary infections in COVID-19 patients, yet it remains unclear whether SARS-CoV-2 infection favors colonization by more virulent or resistant strains. We analyzed 31 nasal *S. aureus* isolates from hospitalized COVID-19 patients to assess antimicrobial resistance, virulence gene content, and genetic diversity. Only two isolates (6.4%) were methicillin resistant, and most strains showed limited resistance beyond the MLSB phenotype. Adhesin genes were highly prevalent, whereas toxin genes were detected in only 16.1% of isolates. *Spa* typing revealed high genetic diversity with no dominant clone. Overall, *S. aureus* isolates from COVID-19 patients did not differ substantially from previously described carriage strains, suggesting no selective enrichment of highly virulent or resistant lineages during SARS-CoV-2 infection.

## 1. Introduction

*Staphylococcus aureus* is a major opportunistic pathogen responsible for a wide spectrum of infections ranging from mild skin disease to severe invasive conditions such as pneumonia, sepsis, and infective endocarditis [1]. Secondary bacterial infections have historically contributed substantially to morbidity and mortality during viral respiratory pandemics, and *S. aureus* remains one of the most frequently reported bacterial pathogens associated with severe viral illness [2,3]. In patients with COVID-19, *S. aureus* has been commonly identified as a cause of bacterial coinfection, particularly among hospitalized and critically ill individuals [4,5,6].

Nasal carriage of *S. aureus* represents a key endogenous reservoir for subsequent infection. Approximately 20–30% of healthy adults are persistent nasal carriers, and carriage is associated with a markedly increased risk of invasive disease, especially in elderly and hospitalized patients [7,8]. In individuals with SARS-CoV-2 infection, reported *S. aureus* carriage rates are comparable to those observed in the general population, yet concerns remain that viral infection, immune dysregulation, or antibiotic exposure may favor colonization by strains with increased virulence or antimicrobial resistance [7,9]. Therefore, detection of *S. aureus* carriage—especially methicillin-resistant *S. aureus* (MRSA)—is important for preventing and managing secondary infections in COVID-19 patients.

The pathogenic potential of *S. aureus* is determined by a combination of antimicrobial resistance, virulence gene content, and genetic background [10,11,12]. Adhesins facilitating colonization and tissue invasion, as well as toxin genes encoding superantigens, contribute to disease severity, while clonal lineage influences transmission dynamics and epidemiology. However, data addressing whether *S. aureus* strains colonizing COVID-19 patients differ from previously described carriage isolates remain limited.

In this Communication, we provide a concise molecular snapshot of nasal *S. aureus* isolates recovered from hospitalized COVID-19 patients, focusing on antimicrobial resistance patterns, selected virulence determinants, and genetic diversity assessed by *spa* typing. The aim of this study was to determine whether SARS-CoV-2 infection is associated with enrichment of particular *S. aureus* clones or virulence profiles among nasal carriers.

## 2. Results

A total of 31 nasal *Staphylococcus aureus* isolates from COVID-19 patients were analyzed. Overall antimicrobial resistance was low. Only two isolates (6.4%) were identified as MRSA, and resistance to antibiotics other than β-lactams was uncommon. The macrolide-lincosamide-streptogramin B (MLSB) resistance phenotype was observed in 35.5% of isolates, predominantly as inducible resistance, while resistance to ciprofloxacin and mupirocin was rare. All isolates were susceptible to glycopeptides, sulfamethoxazole/trimethoprim and aminoglycosides tested (Table 1).

Virulence gene profiling revealed a high prevalence of adhesin genes associated with nasal colonization. Genes encoding laminin-binding protein (eno) and clumping factors (clfA, clfB) were detected in all isolates, whereas other adhesin genes occurred at variable frequencies. In contrast, toxin genes were infrequently detected, with only five isolates (16.1%) carrying genes encoding toxic shock syndrome toxin or enterotoxins. Genes encoding Panton–Valentine leucocidin and exfoliative toxins were absent (Table 2).

**Figure 1:**
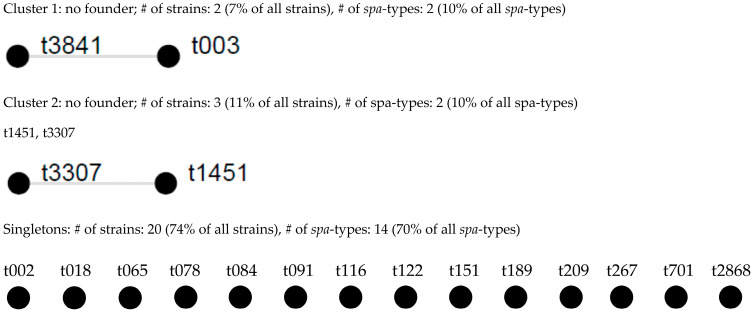
Population structure of 27 *S. aureus* isolates after BURP analysis with a cost of 4. Clusters of linked spa types correspond to spa-CCs. The spa types that were defined as founders of particular clusters are indicated in blue. % of strains based on 27 strains collection; % of spa types based on 20 spa types (including excluded ones); # sign means the word number.

*Spa* typing demonstrated substantial genetic heterogeneity among the isolates, as illustrated in Figure 1. Twenty different *spa* types were identified, with most isolates belonging to unrelated genetic backgrounds and no dominant clone observed. Both MRSA isolates and toxin-positive strains were distributed among distinct *spa* types, indicating the absence of clonal expansion of high-risk lineages among nasal *S. aureus* isolates from COVID-19 patients. Detailed *spa* types and associated resistance and virulence profiles are provided in Supplementary Table S1, while the occurrence of adhesins in relation to *spa* types is presented in Supplementary Table S2.

## 3. Discussion

Secondary bacterial infections, particularly those caused by *Staphylococcus aureus*, have been recognized as important contributors to adverse outcomes during viral respiratory infections, including COVID-19 [13]. However, whether SARS-CoV-2 infection promotes colonization by *S. aureus* strains with enhanced virulence or antimicrobial resistance has remained unclear [14]. In this Communication, we show that nasal *S. aureus* isolates from hospitalized COVID-19 patients do not differ substantially from previously described carriage strains with respect to resistance profiles, virulence gene content, or genetic structure.

Overall antimicrobial resistance among the analyzed isolates was low. Only a small proportion of strains were identified as MRSA, and resistance to non-β-lactam antibiotics was limited, with the MLSB phenotype being the most frequently observed. These findings are consistent with reports from the general population and hospitalized cohorts and do not indicate selective enrichment of multidrug-resistant *S. aureus* during SARS-CoV-2 infection [15,16,17,18,19,20,21,22,23,24]. Importantly, resistance to mupirocin and fluoroquinolones was rare, suggesting that nasal decolonization strategies remain effective in this setting [25].

Analysis of virulence determinants revealed a high prevalence of adhesin genes involved in colonization and host tissue interaction, which is characteristic of *S. aureus* nasal carriage isolates [11,26,27,28]. In contrast, toxin genes encoding superantigens were detected infrequently and were distributed among unrelated genetic backgrounds. The low prevalence of toxin genes and the absence of Panton–Valentine leucocidin or exfoliative toxin genes further support the conclusion that SARS-CoV-2 infection does not preferentially select for highly virulent *S. aureus* strains at the level of nasal carriage [17,29].

Genotyping by *spa* typing demonstrated substantial genetic diversity, with most isolates belonging to unrelated *spa* types and no dominant clone identified. This heterogeneous population structure mirrors that observed in asymptomatic carriers and indicates that colonization in COVID-19 patients arises from diverse endogenous strains rather than expansion of specific epidemic lineages. Although individual isolates carrying notable resistance or virulence traits were identified, these occurred sporadically and were not associated with a particular clonal background [30,31,32,33,34].

This study has limitations, including the relatively small sample size and single-center design, which restrict the generalizability of the findings. Nevertheless, the consistency of our results with existing carriage studies suggests that the observed patterns are representative rather than incidental.

In summary, nasal *S. aureus* isolates from COVID-19 patients exhibit genetic diversity, limited antimicrobial resistance, and virulence profiles comparable to those of previously described carrier strains. These data indicate that SARS-CoV-2 infection is not associated with enrichment of high-risk lineages among nasal carriers, while highlighting the importance of continued surveillance in elderly and hospitalized populations.

## 4. Materials and Methods

### 4.1. Isolation and Identification of S. aureus

Between December 2021 and April 2024, a total of 31 *Staphylococcus aureus* isolates were recovered from anterior nasal swabs of hospitalized patients with confirmed SARS-CoV-2 infection. Samples were collected during routine admission screening at a regional hospital in northern Poland, with one isolate included per patient. Bacterial identification was performed using standard microbiological methods. Ethical approval was obtained from the Local Independent Committee for Ethics in Scientific Research at the Medical University of Gdańsk (NKBBN/525/2021). The main clinical characteristics of the patients are summarized in Supplementary Table S3.

### 4.2. Antimicrobial Susceptibility Testing

Antimicrobial susceptibility was determined using the disk diffusion method in accordance with EUCAST guidelines accurate for the year of specimen’s isolation [35]. Methicillin resistance was identified using cefoxitin disks and confirmed by detection of the PBP2a protein. Inducible macrolide-lincosamide-streptogramin B (MLSB) resistance was assessed using the D-test. Susceptibility to vancomycin and teicoplanin was evaluated using E-test strips. Reference *S. aureus* strains ATCC 25923 and ATCC 43300 were used for quality control.

### 4.3. Detection of Virulence Genes

Genomic DNA was extracted from all isolates, and selected virulence genes encoding adhesins and toxins were detected by PCR using previously described protocols [36,37]. Target genes included adhesins associated with colonization and tissue interaction as well as genes encoding superantigenic toxins.

### 4.4. Spa Typing

Genetic diversity was assessed by *spa* typing based on sequencing of the polymorphic X region of the *spa* gene [38,39]. *Spa* types were assigned using the Ridom SpaServer database, and clonal relatedness was evaluated using the BURP algorithm. Detailed *spa* typing parameters and extended clustering results are provided in the Supplementary Materials.

## Figures and Tables

**Table 1:** Antibiotic resistance of *S. aureus* nasal strains isolated from COVID-19 patients.

Antibiotics	*S. aureus* from COVID-19 Patients *n* = 31 (%)
FOX *	2 (6.4)
MLS_B_	11 (35.5)
iMLS_B_	9 (29.0)
cMLS_B_	2 (6.4)
GEN	0
AN	0
SXT	0
TEI	0
VA	0
MUP	1 (3.2)
CIP	3 (9.7)

FOX—cefoxitin, MLS_B_—macrolide-lincosamide-streptogramin B phenotype resistance (inductive, constitutive), GEN—gentamicin, AN—amikacin, SXT—sulfamethoxazole/trimethoprim, TEI—teicoplanin, VA—vancomycin, MUP—mupirocin, CIP—ciprofloxacin; * positive for PBP2a protein.

**Table 2:** The prevalence of toxin and adhesin genes in *S. aureus* nasal strains isolated from COVID-19 patients.

Virulence Genes	*S. aureus* from COVID-19 Patients *n* = 31 (%)
**Adhesin genes**	
*fib*	27 (87.1)
*eno*	31 (100)
*clfA*	31 (100)
*clfB*	31 (100)
*cna*	14 (45.2)
*fnbB*	10 (32.2)
**Toxin genes**	5 (16.1)
*tst*	2 (6.4)
*sea*	1 (3.2)
*seb*	0
*sec*	1 (3.2)
*sed*	1 (3.2)
*lukS-PV/lukF-PV*	0
*eta*	0
*etb*	0

Genes: *sea* (enterotoxin A), *seb* (enterotoxin b), *sec* (enterotoxin C), *sed* (enterotoxin D); *tst* (toxic shock syndrome toxin-1); *lukS-PV*/*lukF-PV* (Pantone-Valentine leucocidin), *fib* (fibrinogen-binding protein), *eno* (laminin binding protein); *clfA*, *clfB* (clumping factor A, clumping factor B); *fnbB* (fibronectin binding protein B); *cna* (collagen adhesin); *eta* (exfoliatin A), *etb* (exfoliatin B).

## Data Availability

Raw data available per request from the authors.

## Supplementary Materials

The following supporting information can be downloaded at: https://www.mdpi.com/article/10.3390/ijms27031250/s1.
