# Supplementary material for: Nasal Staphylococcus aureus in COVID-19 Patients Shows No Enrichment of High-Risk Clones
Source: Int J Mol Sci. 2026 Jan 27;27(3):1250. doi: 10.3390/ijms27031250 (PMC12897739; doi:10.3390/ijms27031250)
Supplement: Supplementary file 1 [file ijms-27-01250-s001.zip › ijms-4089883-supplementary.pdf]

Supplementary Table S1. *spa* types, clonal complexes, virulence profile and antimicrobial resistance of nasal *S. aureus* isolates from COVID-19 patients.

No of cluster	*Spa* - CC	MLST - CC	Predicted ST	*Spa* type	Virulence profile	Antimicrobial resistance	N
1	NF	-	ST5, ST225	t003	*sed, fib, eno, clfA, clfB*	MRSA- cMLS$_B$	1
1	NF	-	-	t3841	*fib, eno, clfA, clfB*	MSSA	1
2	NF	-	-	t1451	*fib, eno, clfA, clfB*	MSSA- iMLS$_B$	1
2	NF	-	-	t3307	*fib, eno, clfA, clfB, fnbB*	MSSA- iMLS$_B$- CIP	1
					fib, eno, clfA, clfB, fnbB, cna	MSSA- iMLS$_B$	1
S	S	CC5	ST5, ST231	t002	*fib, eno, clfA, clfB, fnbB, cna*	MSSA MUP	1
S	S	CC30	ST30, ST36, ST38	t018	*tst, fib, eno, clfA, clfB, cna*	MSSA- iMLS$_B$	1
S	S	-	ST45, ST46	t065	*fib, eno, clfA, clfB*	MSSA	1
					eno, clfA, clfB, cna	MSSA	1
S	S	-	ST26	t078	*fib, eno, clfA, clfB, fnbB*	MRSA- cMLS$_B$- CIP	1
S	S	-	ST15, ST18	t084	*fib, eno, clfA, clfB, fnbB*	MSSA- CIP	1
					fib, eno, clfA, clfB, fnbB	MSSA	1
S	S	-	ST7	t091	*fib, eno, clfA, clfB, cna*	MSSA	4
					fib, eno, clfA, clfB	MSSA	1
S	S	-	-	t116	*sec, fib, eno, clfA, clfB*	MSSA	1
S	S	-	-	t122	*sea, eno, clfA, clfB*	MSSA- iMLS$_B$	1
S	S	-	-	t151	*eno, clfA, clfB, cna*	MSSA	1
S	S	-	-	t189	*fib, eno, clfA, clfB*	MSSA	1
S	S	-	ST109	t209	*fib, eno, clfA, clfB*	MSSA- iMLS$_B$	1
S	S	-	-	t267	*fib, eno, clfA, clfB, fnbB, cna*	MSSA	1
S	S	-	-	t701	*fib, eno, clfA, clfB, cna*	MSSA	1
S	S	-	-	t2868	*tst, fib, eno, clfA, clfB*	MSSA- iMLS$_B$	1
EC	-	-	-	t3625	*fib, eno, clfA, clfB, fnbB*	MSSA	1
MA	-	-	-	t21923	*fib, eno, clfA, clfB, cna*	MSSA- iMLS$_B$	1
NT	NT			NT	*fib, eno, clfA, clfB, fnbB*	MSSA	1
					fib, eno, clfA, clfB, fnbB, cna,	MSSA	1
					fib, eno, clfA, clfB	MSSA- iMLS$_B$	1
					eno, clfA, clfB, cna	MSSA	1

sea (enterotoxin A), sec (enterotoxin C), sed (enterotoxin D); tst (toxic shock syndrome toxin-1); fib (fibrinogen-binding protein), eno (laminin binding protein); clfA, clfB (clumping factor A, clumping factor B); fnbB (fibronectin binding protein B); cna (collagen adhesin); NF – no founder; S – singleton; EC – excluded; MA – missing alignment; NT – non-typeable, N – number of isolates; MRSA (Methicillin Resistant Staphylococcus aureus); MSSA (Methicillin Sensitive Staphylococcus aureus); MLS$_B$ – macrolide-lincosamide-streptogramin B phenotype resistance (inductive, constitutive); MUP – mupirocin, CIP– ciprofloxacin. .

Supplementary Table S2. Occurrence of adhesin profiles in *S. aureus* COVID-19 isolates in relation to *spa* types.

Adhesin profile	*spa* types	Number of isolates
eno, clfA, clfB	**t122**	1
eno, clfA, clfB, cna	t151, t065, NT,	3
fib, eno, clfA, clfB	**t003**, t3841, t1451, t065, t091, **t116**, t189, t209, **t2868**, NT	10
fib, eno, clfA, clfB, cna	t091, t701, t21923, **t018**	7
fib, eno, clfA, clfB, fnbB	t3307, t078, t084, t3625, NT	6
fib, eno, clfA, clfB, cna, fnbB	t3307, t002, t267, NT	4

fib (fibrinogen-binding protein), *eno* (laminin binding protein); *clfA, clfB* (clumping factor A, clumping factor B); *fnbB* (fibronectin binding protein B); *cna (*collagen adhesin); NT – non-typeable. Bold indicates *spa* types with superantigen genes.

Supplementary Table S3. Clinical characteristics of COVID-19 patients with *Staphylococcus aureus* carriage

Variables	COVID-19 patients n=31
Median age	72.1 (range 40-96)
Gender, male	19 (68.3)
Comorbidities:	
• diabetes mellitus	8 (22.2)
• hypertension	10 (33.3)
• atherosclerosis	2 (6.4)
• solid tumor [a]	2 (6.4)
• renal diseases [b]	3 (9.7)
• cardiac disease [c]	7 (22.6)
• COPD	7 (22.6)
• obesity	5 (18,5)
Laboratory data:	
• C-reactive protein (mg/l), median	103.9
• Procalcitonin (ng/ml), median	4.272
Outcomes<br>• death	3 (9.7)

COPD chronic obstructive pulmonary disease

[a] ovarian tumor, lung tumor

[b] hydrophilic, acute kidney injury

[c] heart failure, atrial fibrillation, coronary artery disease, cardiopathy
